# Assessing the reproducibility of a subject-specific finite element modelling pipeline for the human metastatic vertebrae

**DOI:** 10.1038/s41598-026-46900-4

**Published:** 2026-04-07

**Authors:** Robert Roger, Rajdeep Ghosh, Yuanrui Cai, Fiona Gibson, Áron Lazáry, Lingzhong Guo, Damien Lacroix, Enrico Dall’Ara

**Affiliations:** 1 Insigneo Institute, The University of Sheffield, Sheffield, UK; 2 School of Medicine and Population Health, The University of Sheffield, Sheffield, UK; 3 School of Mechanical, Aerospace and Civil Engineering, The University of Sheffield, Sheffield, UK; 4 School of Electrical and Electronic Engineering, The University of Sheffield, Sheffield, UK; 5 National Center for Spinal Disorders, Budapest, Hungary

**Keywords:** Computed tomography, Metastatic vertebrae, Image segmentation, Subject-specific, Finite element modelling, Reproducibility, Engineering, Medical research

## Abstract

Computed tomography (CT)-based subject-specific finite element (SS-FE) models offer a quantitative approach to assessing the stability of metastatic vertebrae. A critical step in developing SS-FE models is image segmentation, however automatic techniques fail to accurately capture the altered contours of metastatic vertebrae, making manual image segmentation the preferred approach. This introduces operator-dependent steps into the CT-to-FE modelling pipeline that may influence the model’s mechanical properties. To assess these dependencies, this study evaluated intra- and inter-operator reproducibility of a CT-to-FE pipeline applied to six lumbar vertebrae, three radiologically healthy and three lytic metastases. All models were developed using a standardised pipeline incorporating densitometric calibration, 1 mm quadratic tetrahedral meshing, and heterogeneous isotropic elasto-plastic material properties, with vertebrae loaded in uniaxial compression to 1.9% apparent strain. Geometrical/volumetrical metrics from segmented images and mechanical metrics from the SS-FE models were assessed. Intra-operator reproducibility was high across geometric and mechanical metrics, whereas inter-operator reproducibility showed modest variability, particularly in local mechanical metrics of metastatic vertebrae. Consistent volumetric inconsistencies between segmentations significantly correlated with variation in predicted failure load of SS-FE models. Overall reproducibility was high, demonstrating the reliability of the CT-based SS-FE modelling pipeline for estimating vertebral strength.

**Supplementary Information:**

The online version contains supplementary material available at 10.1038/s41598-026-46900-4.

## Introduction

Metastases occur when cancer cells migrate from the primary tumour location through either the bloodstream or lymphatic system and lodge themselves into new tissue. Because of its abundant vascular supply, the spine is a common site for metastatic spread^1^. It is estimated that 16% of all cancer patients are clinically diagnosed with vertebral metastases^2^. With the number of new cancer diagnoses per year predicted to rise globally from 20 million in 2022^3^, to approximately 30 million in 2040^2^, the number of patients presenting with vertebral metastases is expected to significantly increase. Different types of metastases can appear^4^: lytic lesions, associated with focal bone resorption where bone is replaced by cancer cells and fibrous tissue; blastic lesions, associated with a local increase of bone mineral density (BMD); and mixed lesions that include both types of tissue. In particular, lytic lesions are likely to weaken the bone and increase the risk of fracture^5^.

The spinal instability neoplastic score (SINS) is the most common clinical approach for assessing spinal instability. The SINS is based on six criteria evaluated by a clinician: location, mechanical pain, bone lesion quality, spinal alignment, vertebral body collapse and posterolateral involvement of spinal elements^6^. Each assessed metastatic vertebra is given a score between 1 and 18 based on these criteria, with SINS between 1 and 6 suggesting a stable vertebra with no surgical intervention needed, SINS between 7 and 12 would indicate a potentially unstable vertebra with no clear recommendations and SINS between 13 and 18 suggesting an unstable vertebra that requires surgical intervention^7^. Whilst SINS demonstrates high inter-operator reliability and provides clear guidelines on how to proceed for very low and very high SINS, potentially unstable scores have no clear guidelines^8^. This leaves the decision on how to proceed based solely on the clinicians’ experience and with the variety and complexity of cases, it could potentially lead to the overtreatment of patients whose bodies have been weakened by the primary tumour and cancer therapy. Computed tomography (CT)-based subject-specific finite element (SS-FE) models offer a more objective approach to assessing the risk of fracture^9^. SS-FE models based on CT images have been widely used and validated for assessing the mechanical properties of healthy, osteopenic, and osteoporotic vertebrae. Previous studies demonstrated that vertebral SS-FE models derived from CT scans can accurately predict experimental stiffness and failure load under compression^10,11^. Recently, SS-FE models based on subsampled micro-CT images have been demonstrated to accurately predict the structural^12^ and local^13^ properties of human vertebral sections with metastatic lesions.

A critical step in the creation of SS-FE models is the acquisition of the bone geometry through a segmentation process. While there are some automatic segmentation tools based on Convolutional Neural Networks (CNNs) that are developed using both healthy and metastatic vertebrae of all levels^14^, the manual segmentation of the vertebra from CT images is still considered the gold standard. Nevertheless, the changes in BMD and local texture in the vertebra induced by the metastatic lesion, makes it difficult for automatic, semi-automatic and manual techniques to accurately and reproducibly segment the vertebrae. Manual segmentation is still considered as the most reliable method to segment metastatic vertebrae and as the ground truth for the development of automatic algorithms. However, this approach introduced an operator-dependent step in the image segmentation and in the modelling pipeline that may affect the outputs of the models. Therefore, it is paramount to understand the degree to which manual segmentation impacts the assessment of geometrical properties of the metastatic vertebrae and the outputs of biomechanical models used to evaluate their mechanical properties.

Previous geometrical reproducibility studies for vertebrae (Table 1) have employed a wide variety of segmentation techniques from manually segmenting using non-commercial software^15^, to semi-automatic segmentations utilising statistical shape models^16,17^ and fully automatic segmentation employing CNNs^18^. As expected, it has been shown that intra-operator reproducibility is higher than inter-operator reproducibility for numerous metrics. For Dice coefficient (DC) Yao et al.^19^ measured 0.99 ± 0.01 for intra-operator and 0.98 ± 0.01 for inter-operator, whilst Malathy & Nirmala^15^ recorded intra-operator reproducibility of 0.95 ± 0.02 and 3.8 ± 0.6 mm for DC and Hausdorff distance (HD) respectively, with inter-operator reproducibility of 0.92 ± 0.02 for DC and 3.3 ± 0.5 mm for HD. Moreover, Cook et al.^20^ measured a mean surface distance (MSD) of 0.3 mm for both intra- and inter-operator segmentations.

**Table 1:** Summary of previous literature assessing the reproducibility of manual, semi-automatic or automatic image segmentation to assess the geometrical properties of human vertebrae.

**Previous Study**	Stern et al. (2011)^22^	Cook et al. (2012)^20^		Mirzaalian et al. (2013)^16^	Rasoulian et al. (2013)^17^	Malathy & Nirmala (2014)^15^		Yao et al. (2016)^19^				Park et al. (2022)^18^		Khazanchi et al. (2025)^[[21]]^
Anatomical Site	Vertebral body	Vertebrae		Vertebrae	Vertebrae	Vertebrae		Vertebrae				Vertebrae		Vertebrae
Condition	NR	Cadaveric		NR	Healthy	Healthy		Healthy & osteoporotic				Fractured vertebra, caused by trauma, osteoporosis or metastases		Healthy & metastatic
No. of Samples	75	8		154	32	20		17				188		779
Vertebral Level	T1–L5	T12–sacrum		Cervical, thoracic & lumbar	L1–L5	NR		T1–L5				Thoracic & lumbar		Thoracic & lumbar
CT Scan Voxel Size (mm)	1.00	NR		0.78–0.97	0.60–0.90	0.88–1.14		0.31 / 0.35				0.29–0.32		0.31
Segmentation Approach	Semi-automatic	Manual		Semi-automatic	Semi-automatic	Manual		Manual		Semi-automatic	Automatic	Manual	Automatic	Automatic
Segmentation Tool	Deformable model-based segmentation	ScanIP		C + + statistical shape model	Statistical shape model	TURTLESEG		Custom NIH in-house software		Atlas based model	Mean shape model	AsanJ	Convolutional neural network	Meta AI’s segment anything model
No. of Operators	NR	2		1	3	2		2		NR	NR	3		1
Reproducibility Study	Comparison of automatic and manual	Intra	Inter	Comparison of automatic and manual	Comparison of semi-automatic and manual	Intra	Inter	Intra	Inter	Comparison of semi-automatic and manual	Comparison of automatic and manual	Comparison of automatic and manual		Comparison of automatic and manual
Dice Coefficient	NR	NR	NR	NR	NR	0.95 ± 0.02	0.92 ± 0.02	0.99 ± 0.01	0.98 ± 0.01	0.91	0.92	0.93–0.94		0.83 ± 0.05
Hausdorff Distance (mm)	NR	NR	NR	NR	8.9 ± 2.4	3.8 ± 0.6	3.3 ± 0.5	NR	NR	NR	NR	NR		NR
Mean Surface Distance (mm)	1.2 ± 0.3	0.3	0.3	1.4 ± 0.4	1.4 ± 0.6	NR	NR	NR	NR	0.6	0.6	0.4–0.5		NR

NR, not reported.

Other studies compared a semi-automatic or automatic image segmentation technique to manual segmentation to validate the protocol. From these studies the DC range was between 0.83 and 0.94^18,19,21^, HD was measured at 8.9 ± 2.4mm^17^ and MSD ranged between 0.4 and 1.4 mm^16–19,22^.

For mechanical reproducibility of CT-based FE models (Table 2), only one metric has been evaluated, the relative difference (ARD) of the ultimate force (*F*_*U*_). For intra-operator the reported mean difference ranged from 1.8 to 6.4%^23,24^, whilst for inter-operator the mean difference ranged from 5.8 to 17.4%^23,25^. The large differences for the intra-operator assessment may be due to differences in constitutive laws, boundary conditions and meshes in the FE modelling pipelines. Nevertheless, considering that only the reproducibility of a global apparent property as the ultimate force has been assessed in the literature, it is hard to understand if the reproducibility is driven by the local stresses and strains, which are fundamental for the assessment of the mechanical properties of metastatic lesions.

**Table 2:** Summary of previous literature assessing the reproducibility of finite element models based on manual or semi-automatic image segmentation to assess the mechanical properties of human vertebrae.

Previous study	Levillain et al. (2021)^25^	Levillain et al. (2023)^23^				Allard et al. (2024)^[[24]]^	
Anatomical Site	Femur	Vertebral body				Vertebrae	
Condition	Healthy and simulated metastases	Healthy				Healthy (cadaveric)	
No. of Samples	12	21				28	
Vertebral Level	NR	L3				L1–L3	
CT Scan Voxel Size (mm)	0.39	0.33		0.98		0.39	
Segmentation Approach	Manual	Manual				Semi-automatic	
Segmentation Tool	3D Slicer	3D Slicer				ENSAM semi-automatic reconstruction tool	3D Slicer
FE Tool	Ansys	Ansys				Ansys	Ansys
FE Mesh Type	Tetrahedral	Tetrahedral				Hexahedral	Tetrahedral
FE Mesh Order	NR	Quadratic				NR	Quadratic
FE Mesh Size	NR	1 mm				1–1.5 mm	1 mm
FE Material	Heterogeneous, isotropic, linear elastic. Failure defined when 0.33% of total elements exceed their ultimate stress	Heterogeneous, isotropic, nonlinear perfectly elasto-plastic. Yields at 0.7% strain				Heterogeneous, isotropic, linear elastic	Heterogeneous, isotropic, nonlinear perfectly elasto-plastic. Yields at 0.7% strain
FE Loading	5500N applied to femoral head. Distal diaphysis fully restrained	Compressive displacement equivalent to 1.9% apparent strain				Compressive displacement equivalent to 1.5% apparent strain	Compressive displacement equivalent to 1.9% apparent strain
No. of Operators	3	3				2	1
Reproducibility Study	Inter	Intra	Inter	Intra	Inter	Intra	Intra
Ultimate Force Relative Difference (%)	17.4	1.8 ± 1.8	5.8 ± 7.2	3.6 ± 2.7	NR	6.4 ± 6.2	3.5 ± 2.1

NR, not reported.

Moreover, from the current literature it is unclear how the reproducibility in the assessment of geometric/volumetric properties propagates into reproducibility in assessing global and local mechanical metrics with SS-FE models.

The aim of this study is to evaluate the intra- and inter-operator reproducibility and robustness of a CT-based SS-FE modelling pipeline on both the geometrical and biomechanical properties of human vertebrae with and without metastatic lesions.

## Methods

### Overview of the pipeline

**Fig. 1:**
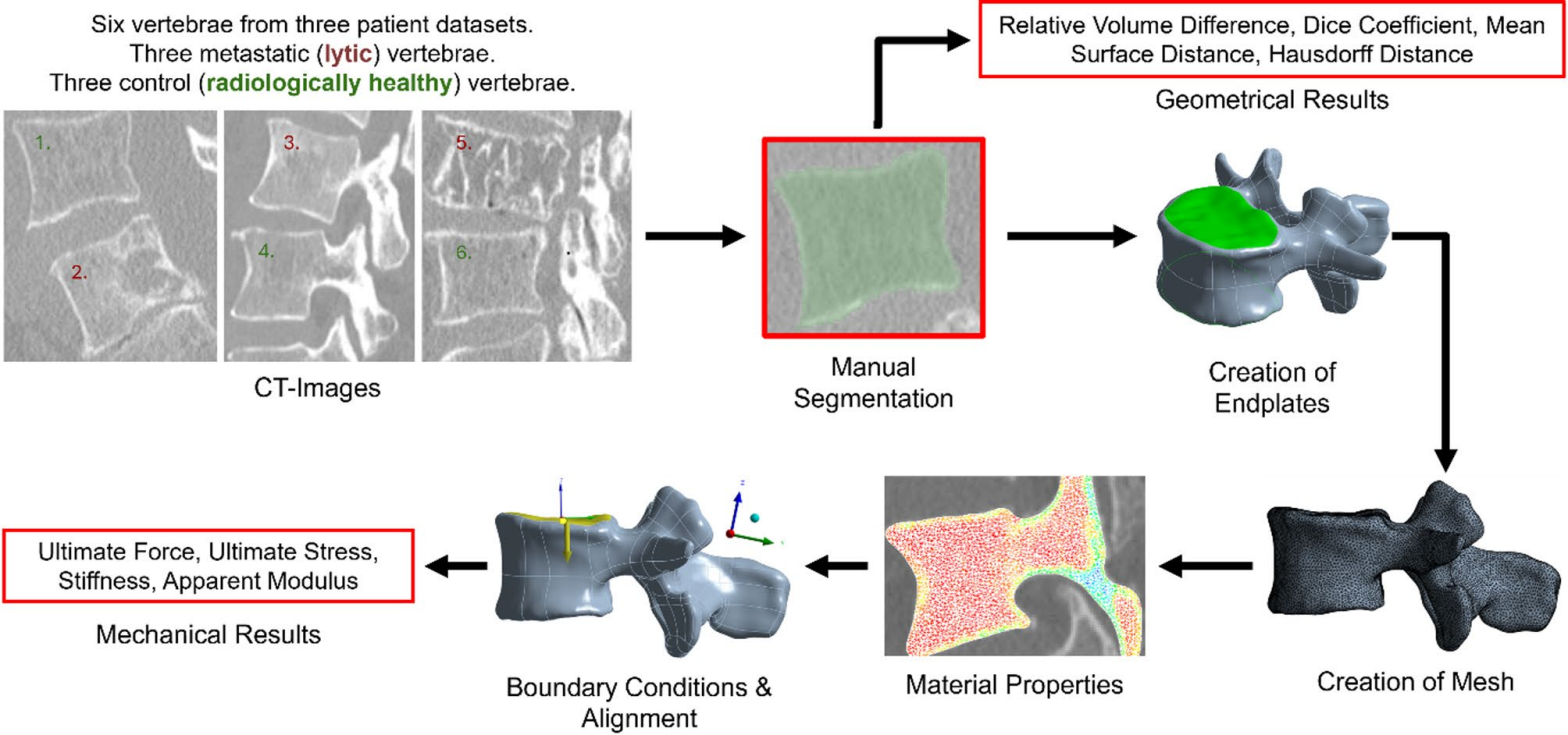
Main steps of the image processing and modelling pipeline workflow. The vertebrae were manually segmented from CT scans. Endplates were identified and used to apply the boundary conditions. A quadratic tetrahedral mesh was created, and nonlinear isotropic heterogeneous material properties were assigned based on the elemental BMD. The FE model was aligned, and uniaxial compression (displacements) was applied. Geometrical and mechanical results were calculated for each segmented image (three intra-operator segmentations, plus two inter-operator segmentations) for each vertebra (N = 6).

In this study a CT-based subject-specific heterogeneous isotropic nonlinear homogenised FE (hFE) model vertebrae with and without metastatic lytic lesion was adapted from a previously published approach^26^. The main parts of the pipeline are highlighted in Fig. 1. Briefly, each vertebra was manually segmented from a CT image (3D Slicer, v5.6.2^27^). Volumetric 1 mm quadratic tetrahedral mesh was used and the nodes of the cortical endplates of the vertebral body were identified to apply boundary conditions. Nonlinear isotropic heterogeneous material properties were assigned using the BMD values of the CT scans after proper densitometric calibration. The hFE model of each vertebra was aligned with a local reference system based on the anatomical planes of the vertebra, and uniaxial compression was applied on the cranial endplate, while the caudal endplate was fixed in all directions. This pipeline was applied to all segmentations created by three operators (three times by Operator-1 for intra-operator reproducibility and another time each by Operator-2 and Operator-3, for inter-operator reproducibility). Geometrical results from the segmented images and mechanical results from the hFE models were compared to evaluate the reproducibility of the pipeline.

### Patient dataset

The anonymised clinical data (quantitative CT (QCT) images of three subjects) used in this study were acquired prospectively at the National Center for Spinal Disorders, Hungary, for a previous study entitled “A multi-center prospective registry for the management and outcome of metastatic spine tumors” (ethical approval Nr: 10848–5/2018/EKU)^9^. The protocol was approved by the Hungarian National Ethical Committee in accordance with national regulations. All patients provided signed informed consent prior to participation, including permission for their pseudo-anonymized data to be evaluated. Six vertebrae were considered, three lytic metastatic vertebrae (one per subject) and three adjacent radiologically healthy vertebrae (one per subject). Vertebrae with lytic lesions of different sizes and located in different areas of the vertebral body were included in the study. More details about the chosen datasets, including primary tumour location and SINS score, are reported in Table 3. The QCT scans were acquired using a Hitachi Presto CT machine (Hitachi Presto, Hitachi Medical Corporation, Tokyo, Japan) with an in-line calibration phantom, 200 mm in length, containing five cylindrical insertion rods (known mean equivalent BMD values of 0.00, 0.05, 0.10, 0.15, and 0.20 g/cm^3^^9^) with a diameter equal to 15 mm, using a scanning protocol that was previously defined by Rijsbergen et al.^28^: voltage of 120 kV, intensity of 225 mA, voxel size of 0.60 × 0.60 × 0.60 mm^3^^9^.

**Table 3:** Details of the datasets included in this study.

Patient	Patient ID	Age	Gender, Male (M) or Female (F)	Primary Tumour	Vertebrae	Condition, Control (C) or Lytic (L)	SINS Score
P2	MV04	63	F	Breast Cancer	L4	C	–
					L5	L	7
P4	MV06	39	F	Colorectal Cancer	L3	L	8
					L4	C	–
P5	MV08	70	F	Aggressive Spinal Haemangioma	L2	L	11
					L3	C	–

### Design of the reproducibility study

One operator (Operator-1) performed three independent segmentations for every vertebra to evaluate the intra-operator reproducibility of the assessment of the geometrical and mechanical properties calculated from the segmented images and the hFE models, respectively. To assess the inter-operator reproducibility two other independent operators (Operator-2 and Operator-3) performed one segmentation each for every vertebra. Operator-1 and Operator-3 had prior experience in manually segmenting CT images of the spine, whilst Operator-2 had no previous experience in manual segmentation. Operator-2 and Operator-3 went through training under the supervision of Operator-1 before performing this study, where two vertebrae were segmented from a training dataset, using the same protocol and software tools as those used in the main study. During this phase, segmentation guidelines and anatomical boundaries were explained and reviewed by Operator-1 to ensure consistency. Practice segmentations were subsequently visually inspected and discussed to confirm adherence to the protocol.

### Segmentation and FE modelling pipeline

Starting from each CT image, the lumbar vertebrae were manually segmented (3D Slicer, v5.6.2^27^). In particular, the contour of each slice in the sagittal plane was conducted from one transverse process to the other. The draw tool was used to contour the outline of the vertebrae and if any mistakes occurred, the eraser tool was used. The size of the eraser tool was adapted according to the shape of the vertebrae. Once the segmentation using the sagittal sections was completed, the segmentation quality was checked in each slice in the transverse and coronal planes. When required, corrections were made, using the draw and eraser tools. Depending on anatomical complexity, the time taken to complete a segmentation approximately ranged from 90 to 180 minutes. Typically segmentations were performed in individual sessions, although operators were able to take breaks when required. After the segmentation was completed, a median 3 mm smoothing filter was applied to reduce irregularities that may arise during manual slice-by-slice contouring, generating smoother surfaces for FE meshing.

To focus on the effect of image segmentation on the hFE outputs, Operator-1 was responsible for continuing the modelling pipeline for each of the provided segmentations for both intra- and inter-operator assessments. The segmentation files were imported into Ansys SpaceClaim (Ansys 2023R2, Synopsis Inc), where the skin surface tool was used to develop a surface model from the faceted data. Superior and inferior endplates were identified and manually labelled using anatomical landmarks on the vertebral body, to develop a surface that the boundary conditions could be applied to.

The created geometries were meshed with quadratic tetrahedral elements with a maximum element size of 1 mm (Ansys Workbench, Mechanical Module, 2023R2, Synopsis Inc), according to the previous mesh refinement study performed by^9^.

The Hounsfield unit (HU) scale in the QCT was converted into BMD values by using a linear densitometric calibration equation. The slope and intercept of the calibration were calculated by fitting a linear regression between the known BMD equivalent values in the five insertions of the phantom, and the average HU values found in a region of interest (prismatic regions with the square section centred within the insertions, with a side length equivalent to half of the diameter of the insertions (7.5 mm)) identified for each insertion (ImageJ, BioFormats function^29^). Each CT scan was densitometrically calibrated individually. The mean BMD in each element was calculated.

The Young’s modulus and yield stress were calculated in each element using Eqs. (1–2) (BONEMAT®, Build_152, 2015^30^) where *ρ*_*ash*_ represents the ash density considered as the ratio between the ash weight and the bulk volume, and *ρ*_*app*_ is the apparent density from the Archimedes’ Principle as the ratio between the wet weight and the bulk volume^31,32^.

1 $${\rho }_{QCT} = {\rho }_{ash} = {\rho }_{app} \times 0.6 [\mathrm{g}/{\mathrm{cm}}^{3}]$$

*E* within Eq. (2) represents the elastic modulus, which is determined through previously published phenomenological law assessed from uniaxial compression and tensile experiments on cylindrical trabecular bone specimens from the human vertebrae^33^.

2 $$E = {4730\rho }_{app}^{1.56} \left[\mathrm{MPa}\right]$$

In Eq. (3), *σ*_*y1*_ represents the isotropic von Mises yield criterion used to model bone plasticity, based on a density-strength relationship^34^.

3 $${\sigma }_{y1}=21.7{\rho }_{app}^{1.52} [\mathrm{g}/{\mathrm{cm}}^{3}]$$

Equation (4) represents an isotropic hardening rule, with a 95% reduction in the post-yield elastic modulus^33–35^.

4 $${E}_{py}=0.05 \times E \left[\mathrm{MPa}\right]$$

The FE models of each vertebra were aligned according to pre-defined anatomical planes. To do so, six virtual landmarks were placed in the most anterior region, and the right and left corners of the posterior region for both the inferior, and superior endplates. These landmarks were then used to create a cranial plane and a caudal plane, which were used to find the bisector plane of the vertebral body. The bisector plane was aligned with the model transverse plane. This procedure was performed solely by Operator-1 to minimise inter-operator variability and was repeated for each model. The resulting rotations showed low variability, with mean standard deviations across vertebrae of 0.36° for flexion–extension, 0.32° for lateral bending, and 0.03° for axial rotation. The alignment of the model was achieved by creating a new coordinate system (Ansys Workbench, 2023R2, Synopsis Inc), where the origin (0,0,0) is located at the centroid of the superior endplate, perpendicular to the endplates.

For each model the vertebral body height (*Hm*) was calculated as the distance between the centroids of the superior and inferior endplates along the axial axis (z). Utilising the binary CT images from the manual segmentation, the cross-sectional area (CSA) was calculated as the mean CSA across each slice of the images, excluding the endplates and posterior elements (3D Slicer, v5.6.2^27^). Due to the small geometric differences between segmentations, the CSA was calculated individually for each model.

A compressive displacement equivalent to 1.9% apparent strain was applied to the nodes of the cranial endplate^10,36^. The nodes of the caudal endplate were fixed in all directions.

The maximum and minimum total principal strains were calculated at each node. Frequency plots were calculated using the strain at each node across the middle 50% height of the vertebral body.

To evaluate local effects of the segmentations, a total of 27 spherical probes with a 2 mm radius were created (Fig. 4). Probe locations were defined relative to each vertebra’s coordinate system created during the alignment procedure, with spheres placed at a consistent distance from the centre of the vertebral body to ensure comparability across vertebrae. Whilst the radius of 2 mm was selected as a compromise between capturing local anatomical variations relevant to mechanical analysis and maintaining robustness to minor segmentation irregularities. For each probe the average strain was calculated.

### Metrics for assessment of reproducibility

#### Geometric/volumetric metrics

The reproducibility of identifying the geometrical and volumetric properties of each vertebra from the manually segmented images was assessed by using the following four metrics (the two segmentations to be compared are referred to as "A" or "B", where B is considered a reference)^37–39^:

1. Absolute relative volume difference (RVD) (Eq. 5), defined as the percentage difference in volume between segmentations.
  5 $$RVD=abs\left(\frac{Volume \left(A\right)-Volume \left(B\right)}{Volume \left(B\right)}\right)$$
2. Dice coefficient (DC) (also known as Dice Similarity Score, Eq. (6)), the measure of the overlap of segmented volumes (0 indicates no overlap, 1 indicates perfect overlap).
  6 $$DC= \frac{2\left|A\cap B\right|}{\left|A\right|+\left|B\right|}$$
3. Mean surface distance (MSD) (Eq. 7), the average distance between corresponding points on the external surfaces of two segmentations. In Eq. (7), *d(a,b)* is defined as the Euclidean distance between boundary pixels a in A and b in B. |A| and |B| represent the number of points on surface A and B respectively.
  7 $$MSD\left(A,B\right)= \frac{1}{2} \left( \frac{1}{\left|A\right|}\sum_{a\in A}\underset{b\in B}{\mathrm{min}} \{ \rm{d} \left(a,b\right) \}+ \frac{1}{\left|B\right|}\sum_{b\in B}\underset{a\in A}{\mathrm{min}}\{d\left(b,a\right)\}\right)$$
4. Hausdorff distance (HD) (Eq. 8), the maximum value of the minimum distance from a point on one segmented surface and a point on the other segmented surface.
  8 $$HD\left(A,B\right)= \mathrm{max}\left\{{\mathrm{max}}  \{ \underset { {a\in A} \ {b\in B}}{\mathrm{min}} \{ \rm{d} \ (a,b)  \}\},{\mathrm{max}} \{ \underset{ \ {b\in B} \ {a\in A}}{\mathrm{min}} \{ \rm{d} \left(b,a\right) \} \} \right\}$$

For each metric the average of the three comparisons (e.g. Operator-1 vs Operator-2, Operator-1 vs Operator-3 and Operator-2 vs Operator-3 for inter-operator analyses) was calculated for both the intra- and inter-operator analyses.

#### Mechanical metrics

The reproducibility associated with the calculation of the mechanical properties from the segmented images was assessed by using the following four metrics^9^:

1. Ultimate force (*F*_*U*_), the resultant axial force at 1.9% imposed apparent deformation.
2. Ultimate stress *(σ*_*U*_) (Eq. 9), ultimate force normalised by the CSA.
  9 $${\sigma }_{U}= \frac{{F}_{U}}{CSA}$$
3. Global apparent stiffness (*K*) (Eq. 10), the slope of the linear range of the force–displacement curves.
  10 $$K= \frac{{F}_{U}}{\Delta l}$$
4. Normalised apparent modulus (*E*_*APP*_) (Eq. 11), stiffness *K* normalised by *Hm* and CSA
  11 $${E}_{APP}= K \times \frac{Hm}{CSA}$$

#### Statistical analyses

For each mechanical metric, mean (m) and standard deviation (SD) were calculated of the intra- and inter-operator analysis, for each vertebra. The coefficient of variation (CV) (Eq. 12) was calculated for each vertebra and the precision error (PE) (Eq. 13) was calculated across the vertebrae^40^.

12 $$CV= \frac{SD}{m}$$

13 $$PE= \sqrt{{\sum }_{j=1}^{n}\frac{{CV}_{j}^{2}}{n}}$$

In Eq. (13), *n* represents the number of models (6 for this study).

The absolute relative difference (ARD) (Eq. 14) was calculated for each mechanical metric for both the intra- and inter-operator analysis^24^.

14 $$ARD=abs\left(\frac{A-B}{\left(\frac{A+B}{2}\right)} \times 100\right)$$

For each geometrical/volumetrical and mechanical metric, the mean values between the intra- and the inter-operator assessments were tested for normality and equal variance before being compared, using a paired *t*-test (statistical level equal to 0.05) (Table 4, Supplementary Table A and Supplementary Table B). Despite the small sample size, linear correlation was used as exploratory investigations to assess potential relationships between geometrical and biomechanical reproducibility metrics.

**Table 4:**
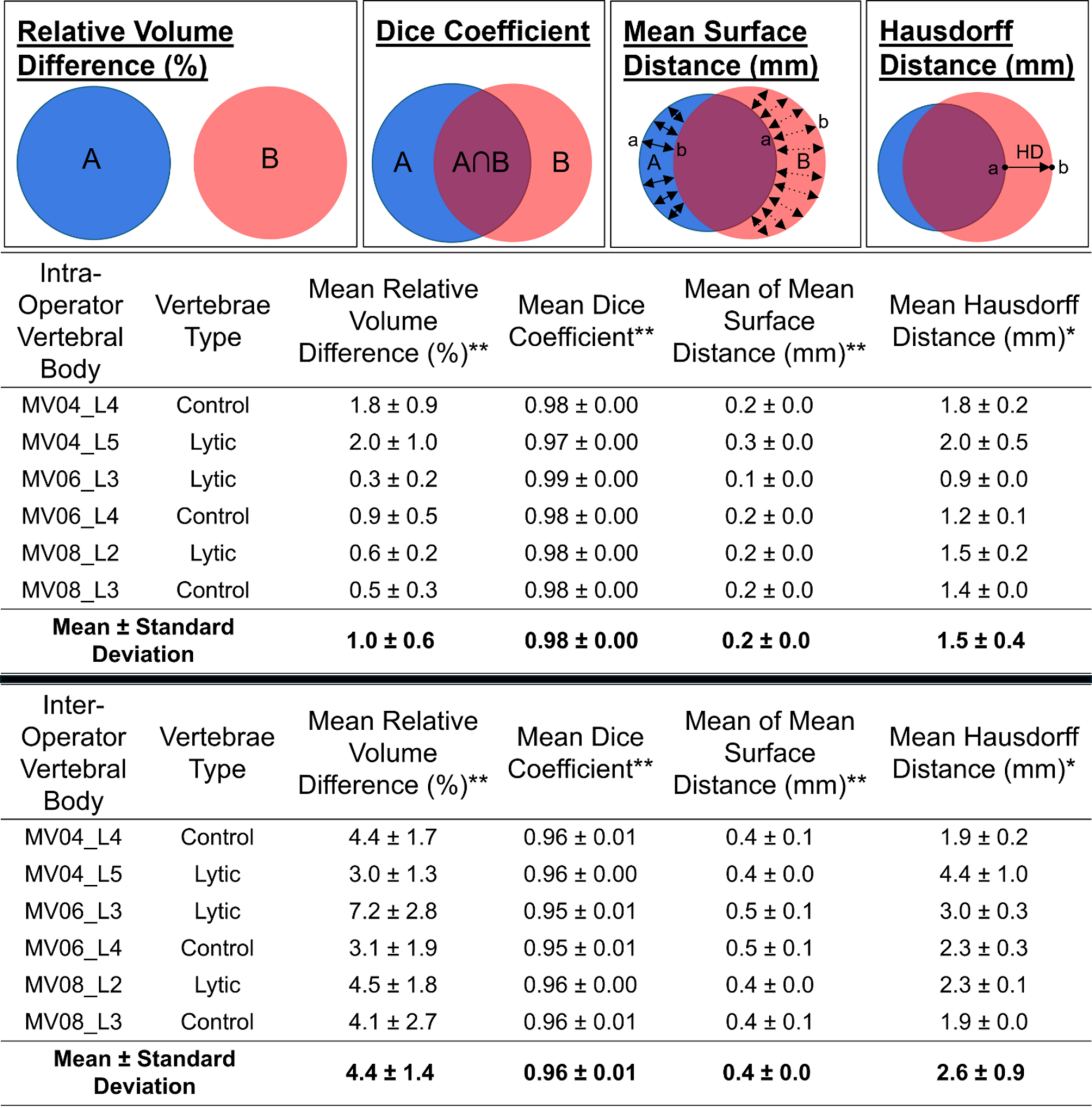
Geometric and volumetric results of the vertebral body for both intra- (top) and inter- (bottom) operator assessments.

Each row is the average and standard deviation for the three comparisons of the vertebra, with the average and standard deviation across all vertebrae in the final row. *, **, ***represent significant differences between the metrics for the intra- and the inter-operator assessments with *p* < 0.05, *p* < 0.01 or *p* < 0.001, respectively.

## Results

The geometric metrics were calculated for both the vertebral body excluding the posterior elements (Table 4), and the whole vertebra (Supplementary Table A).

For the vertebral body geometric and volumetric results indicate a high reproducibility with RVD of 1.0 ± 0.6%, DC of 0.98 ± 0.00, MSD of 0.2 ± 0.0 mm (0.3 ± 0.0 voxels) and HD of 1.5 ± 0.4 mm (2.5 ± 0.7 voxels) when averaged across the mean of each vertebra.

The inter-operator DC indicates a lower reproducibility with RVD of 4.4 ± 1.4%, DC of 0.96 ± 0.01, MSD of 0.4 ± 0.0 mm (0.7 ± 0.0 voxels) and HD of 2.6 ± 0.9 mm (4.3 ± 1.5 voxels) when averaged across the mean of each vertebra.

MV06_L3 was associated with the highest intra-operator reproducibility for all geometrical metrics (RVD of 0.3 ± 0.2%, DC of 0.99 ± 0.00, MSD of 0.1 ± 0.0 mm (0.2 ± 0.0 voxels) and HD of 0.9 ± 0.0 mm (1.5 ± 0.0 voxels)) and, surprisingly, with the lowest inter-operator reproducibility for most geometrical metrics (RVD of 7.2 ± 2.8%, DC of 0.95 ± 0.01, MSD of 0.5 ± 0.1 mm (0.8 ± 0.2 voxels) and HD of 3.0 ± 0.3 mm (5.0 ± 0.5 voxels)). This is likely due to the presence of an osteophyte, with each operator having different interpretations of the contour.

Lytic vertebrae did not exhibit a systematic reduction in reproducibility compared to control vertebrae within the small sample assessed.

For the assessment of the whole vertebra (Supplementary Table A), when averaged across the mean of each vertebra, HD was 2.1 ± 1.0 mm for intra-operator and 6.6 ± 3.3 mm for inter-operator. As these are greater than the values for the vertebral body, this indicates that large errors are localised to the posterior elements.

While the mean bone mineral content (BMC) for all models segmented from the same vertebra remained the same, greater variation was observed in the distributions of elastic moduli assigned to elements in the inter-operator assessments, when compared to those from intra-operator assessments (Supplementary Figure A).

**Fig. 2:**
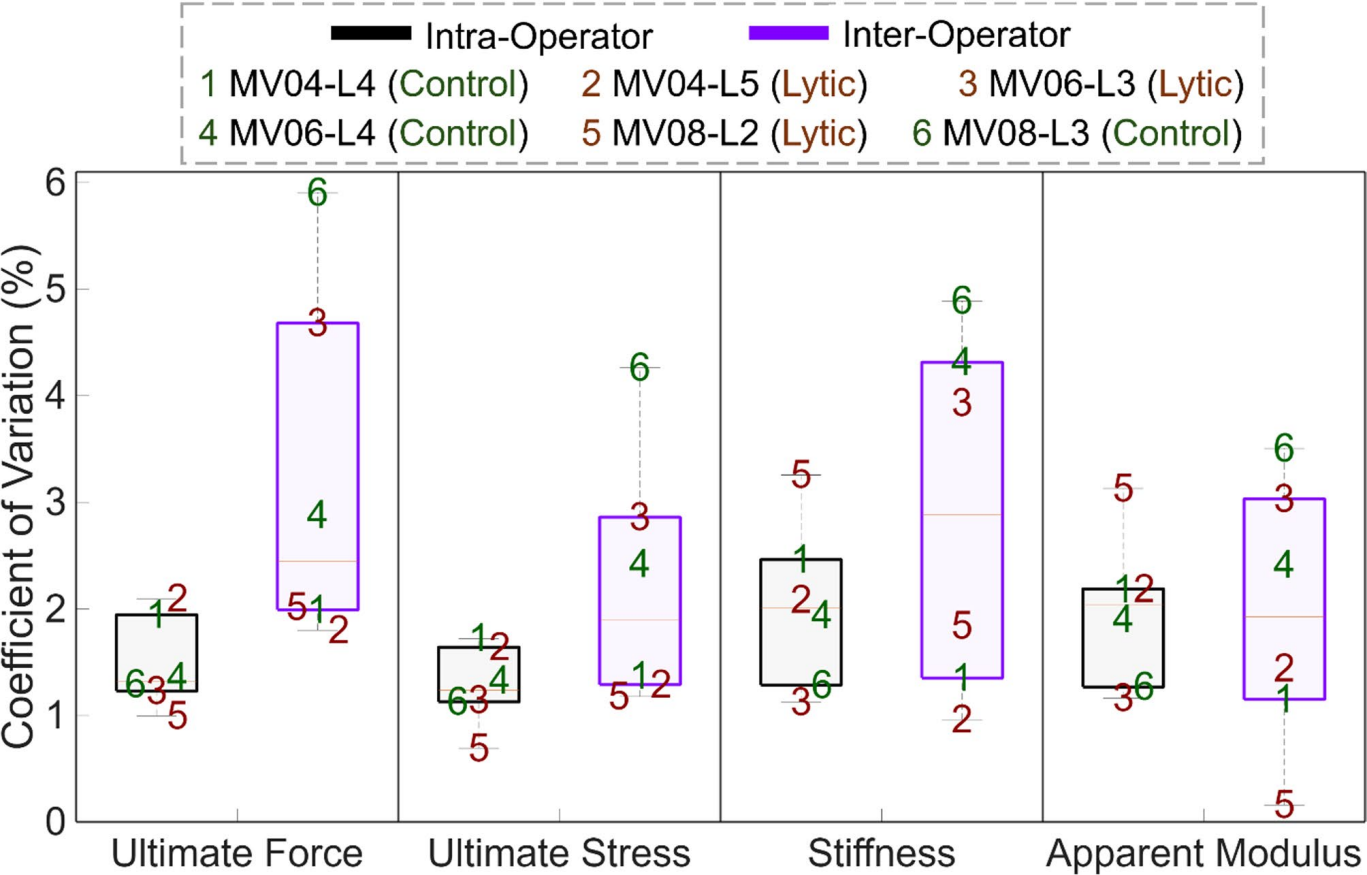
The coefficient of variation of each mechanical metric. Intra-operator is represented by black boxes and inter-operator is represented by blue. Each number represents a different vertebra, with green numbers representing control vertebrae and red representing lytic vertebrae.

For mechanical metrics intra-operator assessments showed lower CV values than inter-operator assessments across all metrics (Fig. 2), although these differences were not statistically significant. In particular, the PE values for *F*_*U*_, *σ*_*U*_, *K* and *E*_*APP*_ were 1.5%, 1.3%, 2.1% and 2.1% for intra-operator and 3.6%, 2.5%, 3.3% and 2.3% for inter-operator, respectively. Details are reported in Supplementary Table B.

Whilst MV08_L3 (labelled as 6 in Fig. 2) was associated with a CV for each mechanical metric that was approximately the lowest quartile for intra-operator assessments, for inter-operator assessments it was consistently associated with the lowest reproducibility.

Load–displacement and stress–strain graphs were created for both intra- and inter-operator assessments (Supplementary Figure B).

**Fig. 3:**
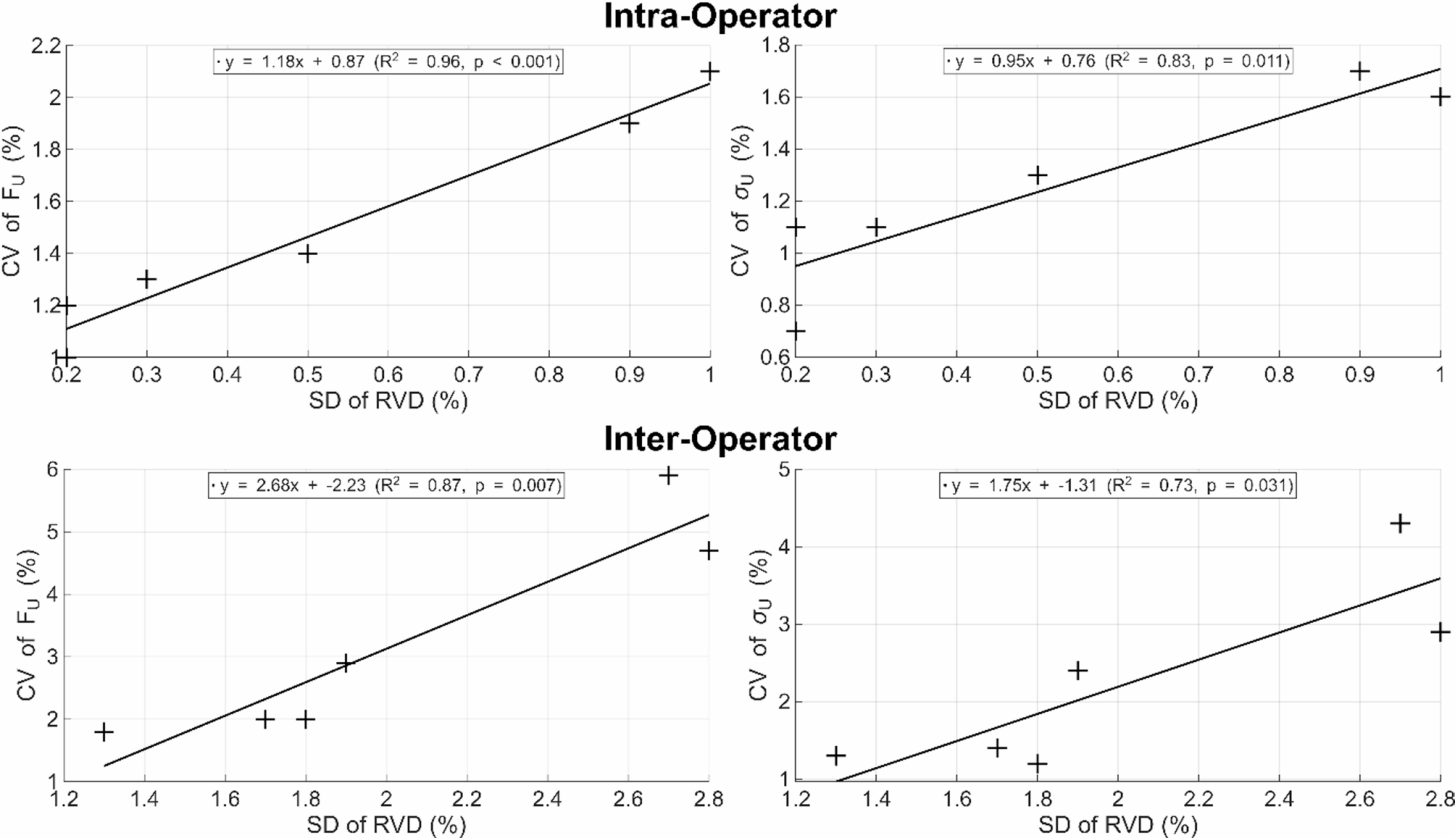
Linear regression of standard deviation of absolute relative volume difference against coefficient of variation of ultimate force (left) and ultimate stress (right), for both intra- (top) and inter- (bottom) operator assessments.

Intra- and inter-operator CV of *F*_*U*_ and *σ*_*U*_ were significantly correlated (*p* < 0.031) with the SD of RVD (intra-operator: R^2^ = 0.96 for *F*_*U*_ and R^2^ = 0.83 for *σ*_*U*_; and R^2^ = 0.87 for *F*_*U*_ and R^2^ = 0.73 for *σ*_*U*_, Fig. 3). While CV of *K* and SD of RVD were significantly correlated (*p* = 0.046) for inter-operator assessments (R^2^ = 0.67), CV of *E*_*APP*_ was not significantly correlated (*p* = 0.07) with the SD of RVD. For intra-operator, neither CV of *K* or *E*_*APP*_ were significantly correlated (*p* > 0.05) with the SD of RVD. The SD of other geometrical metrics did not demonstrate significant correlations (*p* > 0.05) with the CV of the mechanical metrics assessed.

The frequency plots of the minimum and maximum principal strain values (Supplementary Figure C, Supplementary Figure D and Supplementary Figure E) showed greater variations for the inter-operator assessments than for the intra-operator assessments. The variation was greatest in the high frequency, low strain region, between approximately 0 and 0.04 strain for both minimum (ε_p3_) and maximum (ε_p1_) principal strains.

**Fig. 4:**
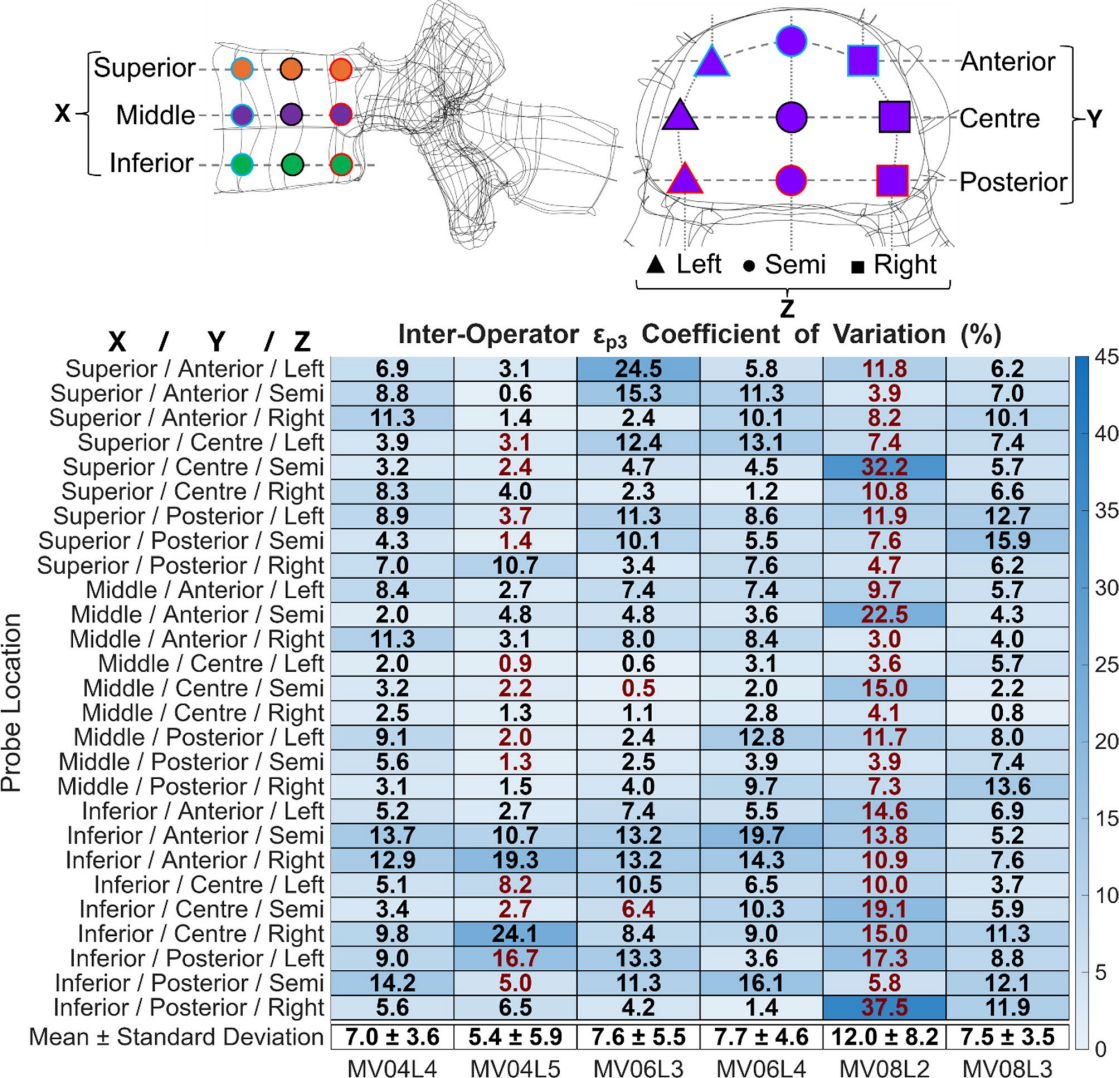
Coefficient of variation of local minimum principal strain (ε_p3_). Each column represents a different vertebra, and each row represents a probes XYZ location (shown in legend). The last row shows the average coefficient of variation across the probes and the standard deviation. The scale of the heatmap is from 0 to 45%. Numbers shown in red represent probes that have elements located within a lesion.

The probes (Fig. 4, Supplementary Figure F and Supplementary Figure G) indicated that the presence of a large lesion within the vertebra reduces the reproducibility of local strain values for inter-operator assessments. For example, for vertebra MV08_L2, for which the whole of vertebral body was affected by a metastatic lesion with a primary tumour of aggressive spinal haemangioma (Table 3), the inter-operator reproducibility for local minimum principal strain, and local maximum principal strain within the probes were 12.0 ± 8.2% and 15.7 ± 9.8%, respectively.

## Discussion

The aim of this study was to evaluate the geometrical and mechanical intra- and inter-operator reproducibility of a CT-based SS-FE modelling pipeline of human vertebrae with or without metastatic lesions, derived from manual image segmentation.

Overall, the study demonstrated high intra-operator reproducibility, for the assessment of both geometrical and mechanical properties. Conversely inter-operator reproducibility was systematically lower. The differences between intra- and inter-operator reproducibility of the segmentation consistently propagated to the mechanical predictions leading to almost double variability (e.g. PE for *F*_*U*_ from 1.5 to 3.6%). The findings indicate that inter-operator dependencies, such as differences in anatomical interpretation and operator experience exert a stronger influence on geometric reproducibility than intra-operator factors such as fatigue. This geometric variability directly affects the mechanical behaviour predicted by the SS-FE models. The relationship between segmentation variability and mechanical reproducibility was particularly evident in the association between the SD of RVD and the CV of *F*_*U*_, suggesting that consistent geometric inconsistencies across the vertebrae can lead to differences in predicted strength. However, as the correlation analyses were exploratory in nature due to the small sample size, this relationship should be considered a preliminary insight into the data, which may inform future studies with larger samples.

The reproducibility observed in this study for geometrical properties of the vertebrae calculated from manual segmentation of the CT scans and mechanical properties estimated from CT-based FE models, aligns well with previous work in the literature.

When compared to previously undertaken manual segmentation reproducibility studies^15,19,20^ the geometrical outputs obtained in this study were similar or improved to those previously reported: for intra-operator DC ranged between 0.95 and 0.99, HD was 3.8 ± 0.6 mm and MSD was 0.3 mm; for inter-operator DC ranged between 0.92 and 0.98, HD of 3.3 ± 0.5 mm and MSD of 0.3. The small differences from this study may be due to the condition of the vertebrae that was segmented. Malathy & Nirmala^15^ and Cook et al.^20^ only segmented healthy vertebrae, whilst Yao et al.^19^ segmented both healthy and osteoporotic vertebrae. The contour of the vertebrae in this study is likely to have been more difficult to visualise due to it being compromised by the lytic lesion (such as with the vertebrae labelled 2 and 5 on the CT images section of Fig. 1). This potentially left more opportunity for operator dependencies such as interpretation and experience to have an effect. Another factor that may impact these geometrical results is the voxel size of the CT scan, with those results at the higher end of the DC range having a voxel size of approximately 0.3 mm and those at the lower end of the DC range approximately 1 mm. With a voxel size of 0.6 mm, the DC values for this study fall within this range. Differences in outputs may also occur due to the application of smoothing filters. By applying smoothing (e.g. 3 mm median smoothing in the current study), local discrepancies between segmentations may be reduced, improving the intra-/inter-operator reproducibility. Cook et al.^20^ segmented CT-images of lumbar vertebrae and applied a recursive Gaussian filter to the mask, and for both intra- and inter-operator a MSD of 0.3 mm was observed, which is greater than that of the intra-operator MSD observed in the present study (0.2 ± 0.0 mm for both vertebral body only, excluding the posterior elements, and whole vertebra), but lower than that observed for inter-operator MSD in the present study for both the vertebral body and whole vertebra (0.4 ± 0.0 mm and 0.5 ± 0.0 mm respectively). Malathy & Nirmala^15^ segmented CT-images of vertebrae, but did not apply any smoothing filter and observed an intra-operator HD of 3.8 ± 0.6 mm and an inter-operator HD of 3.3 ± 0.5 mm. With no smoothing applied the intra-operator variability for HD was found to be approximately double of that observed in this study (1.5 ± 0.4 mm for vertebral body only and 2.1 ± 1.0 mm for the whole vertebra). However for inter-operator variability, HD was greater than that observed in the present study for the vertebral body only (2.6 ± 0.9 mm) but was half that of the whole vertebra (6.6 ± 3.3 mm).

It was expected that due to changes in BMD and local texture in the vertebra induced by the metastatic lesion, manual segmentation would show higher reproducibility than automatic techniques. Park et al.^18^ and Khazanchi et al.^21^ both compared an automatic and manual techniques applied to datasets that included metastatic vertebrae. Whilst both studies had voxel sizes roughly half of the voxel size of this study, the MSD was approximately double^18^ and the DC was up to 0.13 lower^21^ than that found for the inter-operator analysis in this study, showing that manual segmentation has greater reproducibility.

From a mechanical perspective, previous studies have examined the reproducibility of FE models of healthy vertebrae derived from manual and semi-automatic segmentations^23,24^. Levillain et al.^23^ reported an intra-operator ARD of the failure load of 1.8 ± 1.8%, which is three times greater than the present study, for manual segmentations performed on images with a voxel size of 0.33 mm, as opposed to voxel size of 0.60 mm in this study. Whilst Allard et al.^24^ reported intra-operator ARD of the failure load of 3.5 ± 2.1%, which is six times greater than the present study, for semi-automatic segmentations performed on images with a voxel size of 0.39 mm. Both studies utilised the same segmentation and FE tool, FE mesh and FE loading conditions as the present study; however differences in reproducibility may stem from the material properties applied This study used heterogeneous, isotropic, nonlinear elasto-plastic material properties with a 95% reduction in post-yield elastic modulus, whereas both previous studies used heterogeneous, isotropic, nonlinear perfectly elasto-plastic properties that yielded at 0.7% strain without isotropic hardening. Levillain et al.^23^ had the same number of operators (three) which also included a range of previous experience. They reported that the variability in predicted failure load increased when comparing the expert-beginner pair relative to the expert-competent pair, highlighting the influence of operator experience on mechanical outcomes. This reflects the present study, in which MSD approximately doubled between intra- and inter-operator analyses. Whilst focusing on a different anatomical site, the femur, Levillain et al.^25^ examined both healthy and metastatic bone. The ARD of failure load for inter-operator was reported as 17.4% which is much greater than the inter-operator results for the vertebrae (5.8 ± 7.2% from Levillain et al.^23^, 5.1 ± 2.6% in this study, Supplementary Table C). The voxel size is not the only difference between this study and Levillain et al.^25^ as they use heterogeneous, isotropic, linear elastic material properties with failure defined as 0.33% of element exceeding their ultimate stress, when applied with a load of 5500N. Such a failure criterion is likely responsible for the larger variability in ultimate force ARD, as it is more sensitive to operator-dependent segmentation and meshing differences than models loaded to failure.

Overall, the present findings corroborate earlier work demonstrating that manual segmentation can achieve high reproducibility for vertebral FE modelling, particularly when performed by trained operators. However, the persistence of inter-operator uncertainties highlights the need for standardised segmentation protocols and quality-control procedures to ensure consistent geometric definitions across operators.

The strong agreement observed between geometric and mechanical reproducibility reinforces the robustness of the CT-based SS-FE modelling pipeline. The clear correlation between geometric deviations and mechanical variability indicates that small differences in segmentation propagate predictably through the modelling workflow. This relationship highlights the importance of precise and consistent segmentation, particularly for studies or clinical applications aiming to use SS-FE models to assess vertebral strength or fracture risk.

Some limitations should be considered when interpreting the results. First, the sample size was limited to six vertebrae, segmented by three operators; while sufficient to quantify reproducibility trends, a larger cohort including multiple spinal levels would better generalise the findings. Second, the creation of endplates was performed by the same operator for all models, failing to capture the full spectrum of uncertainties present in an end-to-end clinical workflow. However, this choice was taken to focus on the variability induced by the segmentation operation on the final results. Lastly, the present dataset only represents one type of metastases, lytic. While this is the most critical type of lesions with respect to reduction of bone strength, other lesion types, such as blastic or mixed, may exhibit different density patterns and structural characteristics that could influence both segmentations and FE outputs. Future studies will be performed to generalise the findings to a broader range of lesion types.

In conclusion, this study quantified both intra- and inter-operator reproducibility of a CT-based subject-specific FE modelling pipeline derived from manual vertebral segmentation. The results demonstrate that intra-operator segmentation is highly reproducible, while inter-operator differences produce modest but systematic variability in both measured geometrical properties and predicted mechanical properties. These findings reinforce the reliability of manually derived SS-FE models for vertebral strength estimation and provide a quantitative basis for benchmarking future developments in automated segmentation and clinical FE modelling workflows.

## Supplementary Information

Below is the link to the electronic supplementary material.

Supplementary Material 1

## Data Availability

The clinical datasets generated and/or analysed during the current study are not publicly available due to the current ethical approval. However, the secondary data generated in this study are available from the corresponding author on reasonable request.
