# Supplementary material for: Assessing the reproducibility of a subject-specific finite element modelling pipeline for the human metastatic vertebrae
Source: Sci Rep. 2026 Apr 7;16:16092. doi: 10.1038/s41598-026-46900-4 (PMC13199536; doi:10.1038/s41598-026-46900-4)
Supplement: Supplementary file 1 — Supplementary Material 1 [file 41598_2026_46900_MOESM1_ESM.pdf]

Supplementary Materials

*Supplementary Table A: Geometric and volumetric results of the whole vertebra for both intra- (top) and inter- (bottom) operator assessments. Each row is the average and standard deviation for the three comparisons of the vertebra, with the average and standard deviation across all vertebrae in the final row. *, **, *** represent significant differences between the metrics for the intra- and the inter-operator assessments with p<0.05, p<0.01 or p<0.001, respectively.*

Supplementary Table A

Intra-Operator	Vertebrae Type	Mean Relative Volume Difference (%) *	Mean Dice Coefficient ***	Mean of Mean Surface Distance (mm) *	Mean Hausdorff Distance (mm) *
MV04_L4	Control	2.5 ± 1.3	0.98 ± 0.00	0.2 ± 0.0	1.8 ± 0.2
MV04_L5	Lytic	2.0 ± 0.9	0.97 ± 0.01	0.2 ± 0.0	2.4 ± 0.5
MV06_L3	Lytic	0.6 ± 0.4	0.98 ± 0.00	0.1 ± 0.0	1.3 ± 0.1
MV06_L4	Control	2.0 ± 1.0	0.98 ± 0.00	0.2 ± 0.0	1.3 ± 0.2
MV08_L2	Lytic	0.4 ± 0.2	0.97 ± 0.00	0.2 ± 0.0	1.5 ± 0.2
MV08_L3	Control	0.6 ± 0.3	0.97 ± 0.00	0.2 ± 0.0	4.0 ± 0.5
**Mean ± Standard Deviation**		**1.4 ± 0.8**	**0.98 ± 0.01**	**0.2 ± 0.0**	**2.1 ± 1.0**

Inter-Operator	Vertebrae Type	Mean Relative Volume Difference (%) *	Mean Dice Coefficient ***	Mean of Mean Surface Distance (mm) *	Mean Hausdorff Distance (mm) *
MV04_L4	Control	4.5 ± 1.7	0.94 ± 0.02	0.5 ± 0.1	8.0 ± 5.3
MV04_L5	Lytic	3.0 ± 2.1	0.94 ± 0.01	0.5 ± 0.1	12.0 ± 5.4
MV06_L3	Lytic	7.9 ± 3.7	0.93 ± 0.02	0.5 ± 0.1	7.0 ± 2.4
MV06_L4	Control	5.0 ± 1.9	0.94 ± 0.02	0.5 ± 0.1	2.5 ± 0.4
MV08_L2	Lytic	6.4 ± 2.7	0.94 ± 0.00	0.5 ± 0.0	4.2 ± 1.2
MV08_L3	Control	7.7 ± 4.7	0.93 ± 0.01	0.5 ± 0.1	6.1 ± 3.0
**Mean ± Standard Deviation**		**5.7 ± 1.7**	**0.94 ± 0.01**	**0.5 ± 0.0**	**6.6 ± 3.3**

Supplementary Figure A

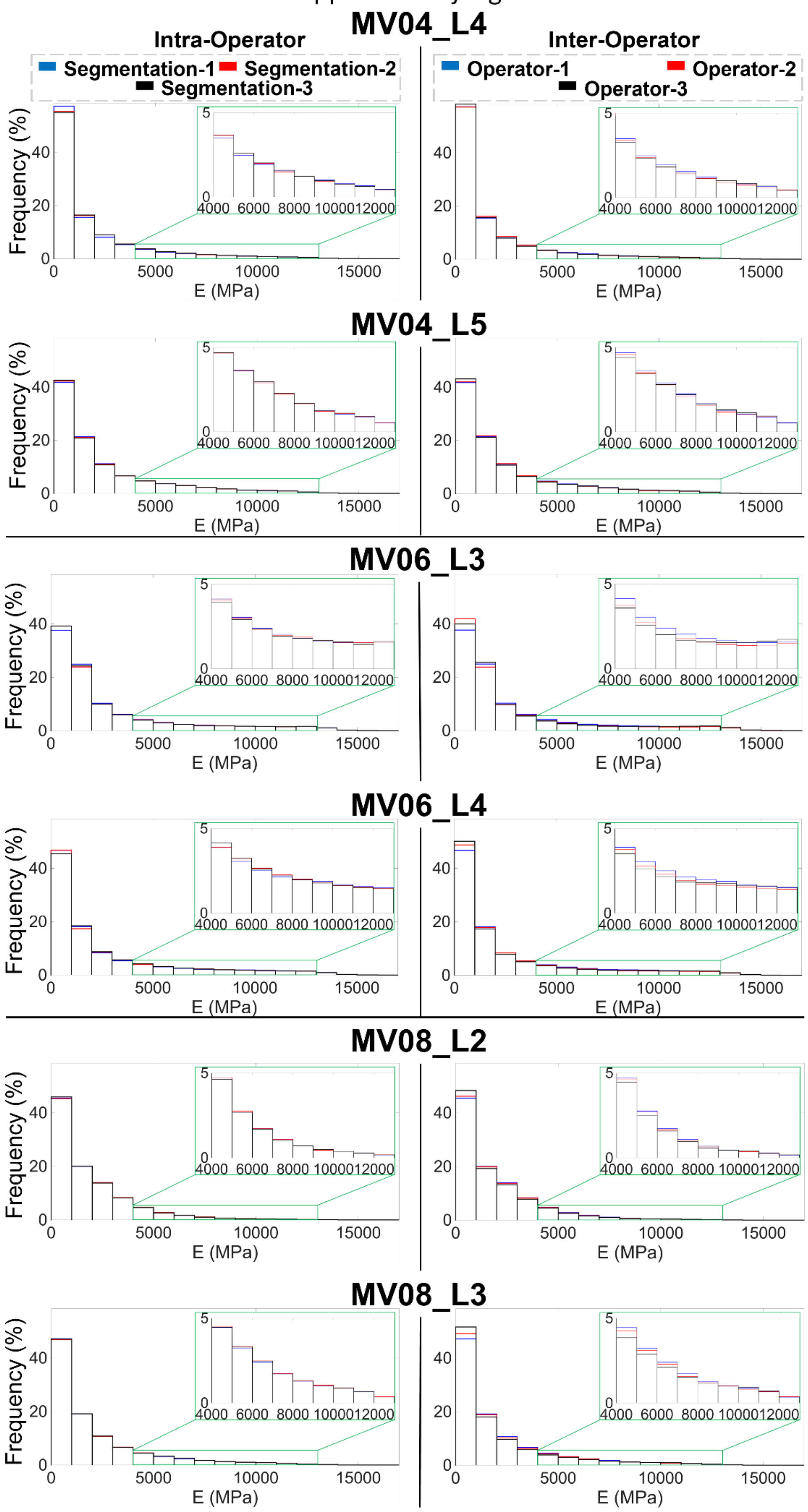

Supplementary Figure A: Frequency plots of elastic modulus assigned to elements for patient dataset MV04 (top), MV06 (middle) and MV08 (bottom)*. Bin width is 1000 MPa. Intra-operator is on the left and inter-operator is on the right.*

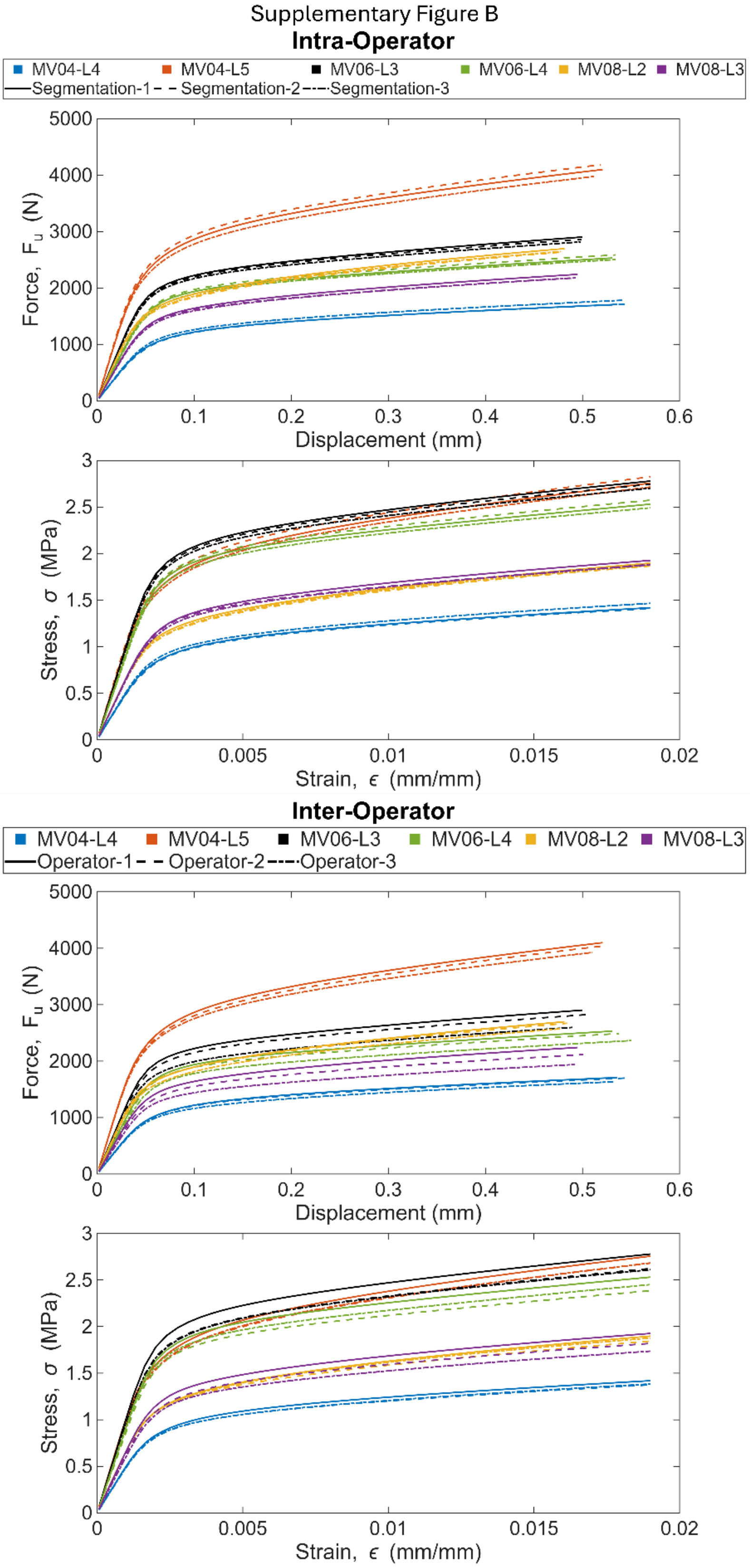

Supplementary Figure B: Force-displacement and stress-strain graphs for intra- (top) and inter-operator (bottom) assessments

Supplementary Figure C

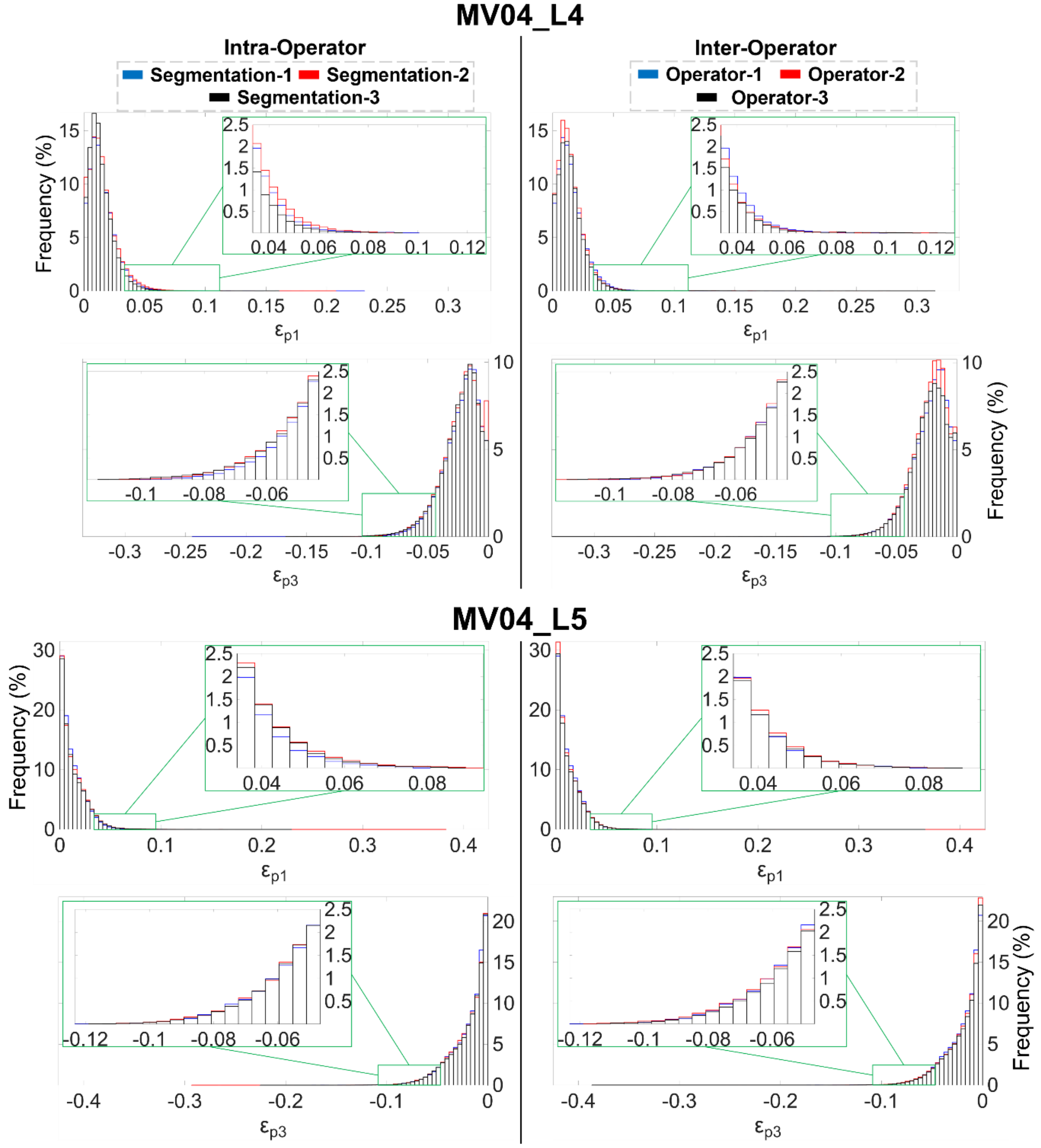

Supplementary Figure C: Frequency plots of the minimum and maximum total principal strain for both intra- and inter-operator for patient dataset MV04, L4 (top) and L5 (bottom). Bin width is 1% of the highest value of strain on the vertebra, 0.3 for L4 and 0.4 for L5. Intra-operator is on the left and inter-operator is on the right. For each vertebra maximum principal strain (ε_{p1}) is on the top and minimum principal strain (ε_{p3}) is on the bottom. The zoom in (green square) shows strain values that have a frequency of between 0.01% and 2.5%.

Supplementary Figure D

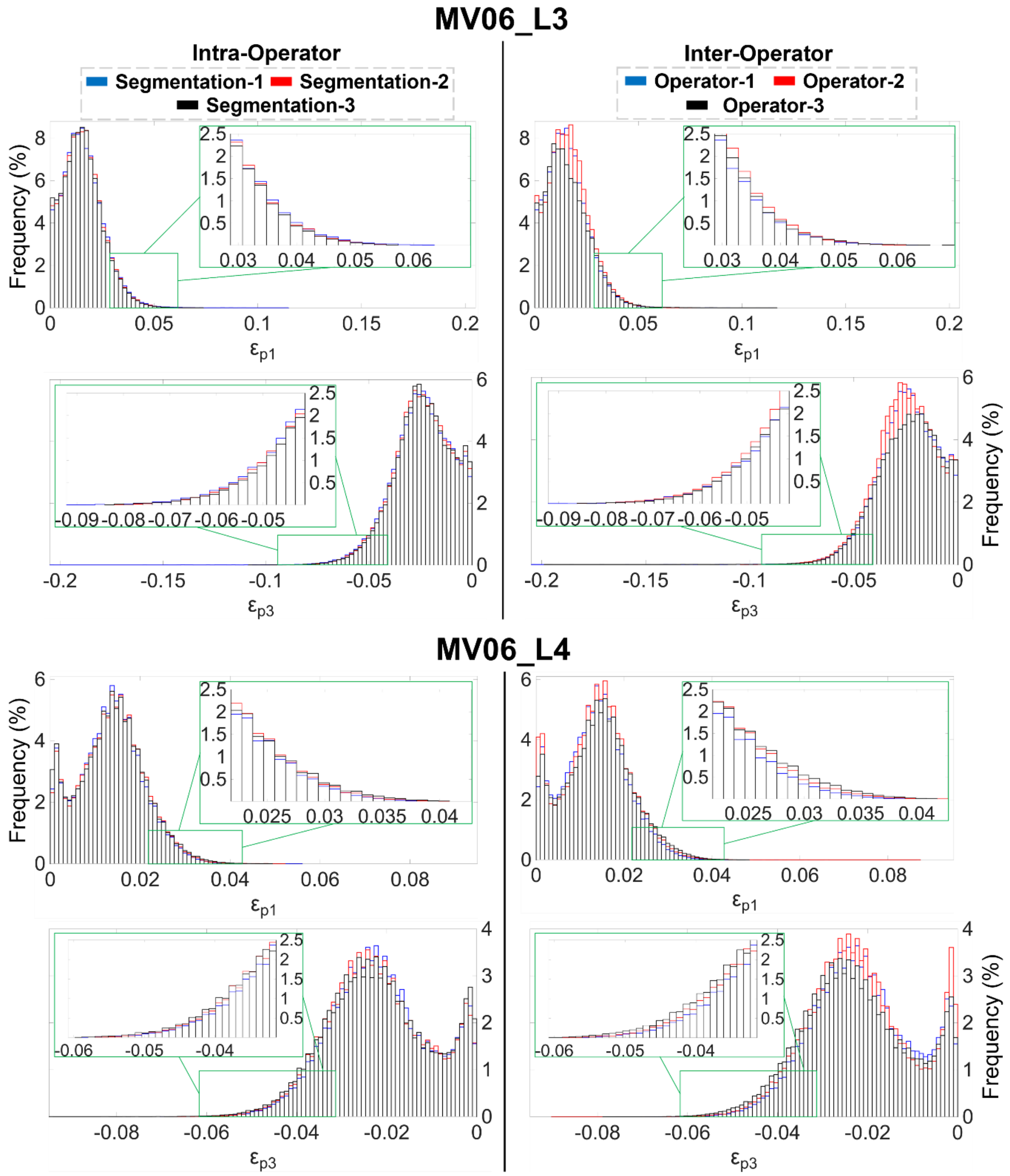

Supplementary Figure D: Frequency plots of the minimum and maximum total principal strain for both intra- and inter-operator for patient dataset MV06, L3 (top) and L4 (bottom). Bin width is 1% of the highest value of strain on the vertebra, 0.2 for L3 and 0.09 for L4. Intra-operator is on the left and inter-operator is on the right. For each vertebra maximum principal strain (ε_{p1}) is on the top and minimum principal strain (ε_{p3}) is on the bottom. The zoom in (green square) shows strain values that have a frequency of between 0.01% and 2.5%.

Supplementary Figure E

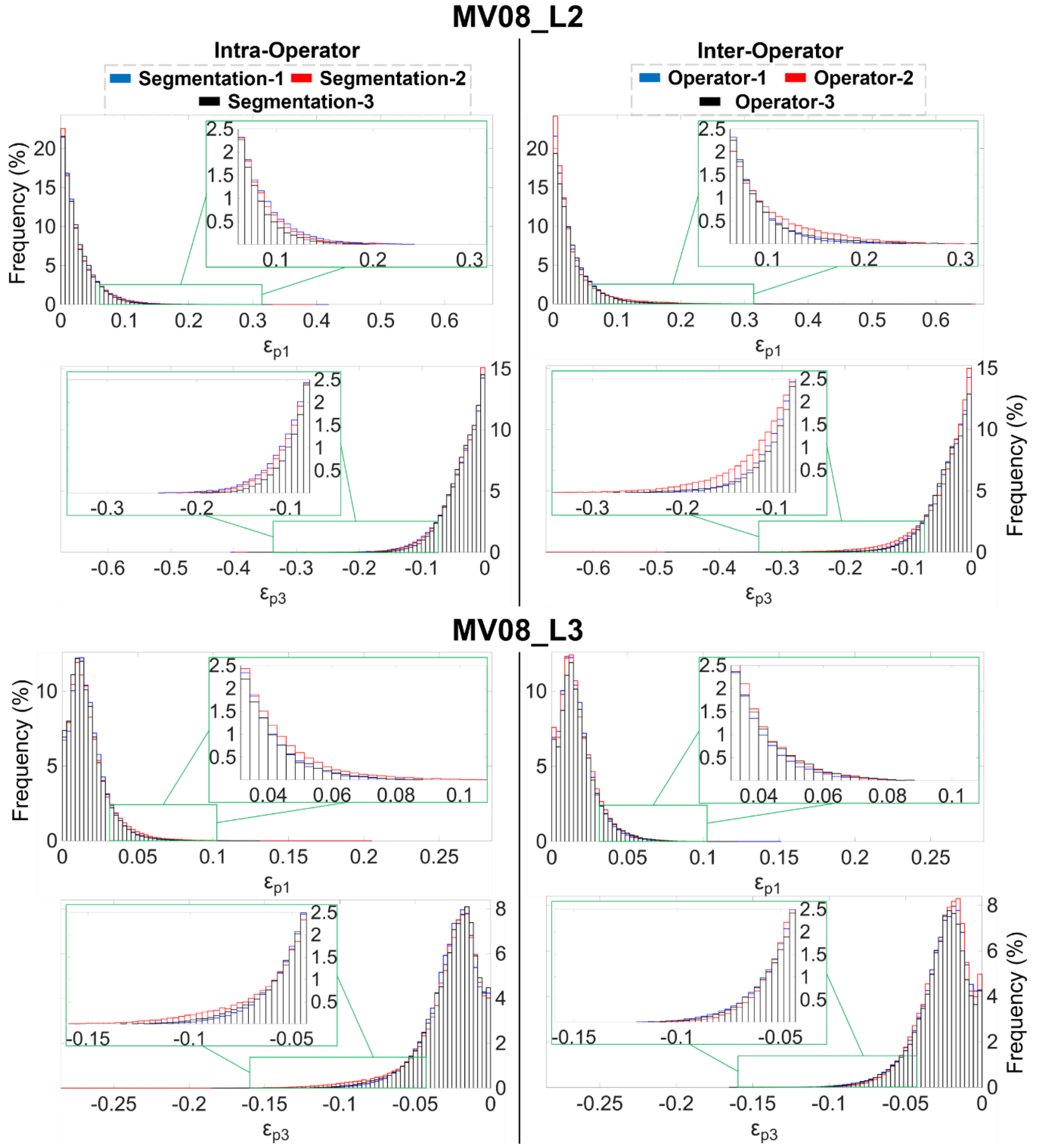

Supplementary Figure E: Frequency plots of the minimum and maximum total principal strain for both intra- and inter-operator for patient dataset MV08, L2 (top) and L3 (bottom). Bin width is 1% of the highest value of strain on the vertebra, 0.62 for L2 and 0.3 for L3. Intra-operator is on the left and inter-operator is on the right. For each vertebra maximum principal strain (ε_{p1}) is on the top and minimum principal strain (ε_{p3}) is on the bottom. The zoom in (green square) shows strain values that have a frequency of between 0.01% and 2.5%.

Supplementary Figure F

Intra-Operator ε_{p1} Coefficient of Variation (%)

Probe Location (X / Y / Z)	MV04L4	MV04L5	MV06L3	MV06L4	MV08L2	MV08L3
Superior / Anterior / Left	7.3	1.1	26.0	4.7	3.3	0.4
Superior / Anterior / Semi	7.9	3.5	11.7	7.3	6.1	3.8
Superior / Anterior / Right	16.1	0.5	7.9	2.9	3.2	5.4
Superior / Centre / Left	7.1	3.6	13.1	10.9	7.4	15.1
Superior / Centre / Semi	12.3	0.6	2.3	2.0	4.0	12.6
Superior / Centre / Right	21.4	4.0	3.9	4.5	6.4	9.0
Superior / Posterior / Left	10.3	12.8	3.5	12.3	2.4	8.5
Superior / Posterior / Semi	23.5	1.5	1.6	7.4	13.6	12.5
Superior / Posterior / Right	25.0	11.3	5.2	9.2	8.8	4.1
Middle / Anterior / Left	7.4	8.2	2.8	1.8	0.8	6.6
Middle / Anterior / Semi	9.8	9.8	1.1	3.2	9.1	4.1
Middle / Anterior / Right	6.7	2.4	3.8	1.8	2.6	4.8
Middle / Centre / Left	9.8	4.9	0.7	3.9	4.4	10.8
Middle / Centre / Semi	11.2	4.2	1.2	1.4	3.1	5.3
Middle / Centre / Right	5.9	4.3	0.9	2.0	4.9	2.2
Middle / Posterior / Left	15.0	7.9	1.6	5.9	4.2	3.4
Middle / Posterior / Semi	4.6	2.2	3.1	4.4	2.1	14.3
Middle / Posterior / Right	3.7	2.9	3.7	2.1	3.9	3.0
Inferior / Anterior / Left	5.9	5.5	11.6	6.6	13.7	2.1
Inferior / Anterior / Semi	11.1	11.3	8.6	22.2	6.5	2.8
Inferior / Anterior / Right	18.4	26.0	9.4	4.5	3.7	5.4
Inferior / Centre / Left	15.9	6.0	10.3	3.5	14.2	11.9
Inferior / Centre / Semi	3.6	15.3	12.3	6.2	18.4	5.9
Inferior / Centre / Right	7.6	6.3	5.5	2.5	4.9	23.4
Inferior / Posterior / Left	21.1	11.1	4.0	7.8	6.5	1.9
Inferior / Posterior / Semi	18.6	8.2	13.4	8.3	14.1	7.6
Inferior / Posterior / Right	20.6	7.2	7.2	3.8	13.9	6.3
Mean ± Standard Deviation	12.2 ± 6.3	6.8 ± 5.4	6.5 ± 5.6	5.7 ± 4.3	6.9 ± 4.6	7.2 ± 5.1

Intra-Operator ε_{p3} Coefficient of Variation (%)

Probe Location (X / Y / Z)	MV04L4	MV04L5	MV06L3	MV06L4	MV08L2	MV08L3
Superior / Anterior / Left	3.0	2.1	21.7	4.1	2.4	0.6
Superior / Anterior / Semi	6.1	2.1	11.5	3.7	3.9	2.3
Superior / Anterior / Right	6.1	0.3	7.4	2.7	3.9	3.5
Superior / Centre / Left	3.2	2.2	13.6	7.2	10.7	14.6
Superior / Centre / Semi	5.6	1.9	2.3	4.3	5.8	12.7
Superior / Centre / Right	9.0	4.3	3.3	3.1	7.1	5.0
Superior / Posterior / Left	7.3	9.2	3.9	13.7	1.7	8.4
Superior / Posterior / Semi	18.5	1.4	0.9	2.8	9.6	12.0
Superior / Posterior / Right	19.7	10.6	5.4	9.2	10.7	3.2
Middle / Anterior / Left	6.8	7.0	2.5	2.0	2.6	5.6
Middle / Anterior / Semi	6.4	6.0	1.1	1.4	7.2	2.3
Middle / Anterior / Right	5.3	1.2	4.6	1.6	2.2	3.1
Middle / Centre / Left	7.3	1.8	1.2	3.9	2.6	3.1
Middle / Centre / Semi	4.6	4.8	1.3	1.3	1.3	4.2
Middle / Centre / Right	4.9	1.6	1.4	1.4	3.4	2.6
Middle / Posterior / Left	13.0	7.0	1.5	7.6	3.1	5.4
Middle / Posterior / Semi	7.8	1.5	1.9	4.7	1.3	19.1
Middle / Posterior / Right	8.0	0.8	2.6	2.7	6.8	3.8
Inferior / Anterior / Left	1.6	7.6	10.1	5.3	13.0	1.9
Inferior / Anterior / Semi	9.0	8.6	6.9	12.0	3.0	1.1
Inferior / Anterior / Right	17.3	18.4	6.7	3.2	4.1	4.0
Inferior / Centre / Left	4.1	5.9	8.1	3.5	11.1	8.6
Inferior / Centre / Semi	3.1	2.2	7.8	4.3	8.7	3.0
Inferior / Centre / Right	17.3	4.2	5.8	1.4	5.9	12.0
Inferior / Posterior / Left	17.9	15.0	2.8	7.3	4.8	0.7
Inferior / Posterior / Semi	11.6	5.7	12.5	6.6	11.6	5.7
Inferior / Posterior / Right	18.5	4.5	6.0	3.8	14.3	6.3
Mean ± Standard Deviation	9.0 ± 5.5	5.1 ± 4.3	5.7 ± 4.8	4.6 ± 3.1	6.0 ± 3.8	5.7 ± 4.6

Supplementary Figure F: Coefficient of variation of local maximum principal strain (ε_{p1}) (top) and local minimum principal strain (ε_{p3}) (bottom). Each column represents a different vertebra, and each row represents a probes XYZ location (shown in legend). The last row shows the average coefficient of variation across the probes and the standard deviation. The scale of the heatmap is from 0% to 45%. Numbers shown in red represent probes that have elements located within a lesion.

Supplementary Figure G

Inter-Operator ε_{p1} Coefficient of Variation (%)

Probe Location (X / Y / Z)	MV04L4	MV04L5	MV06L3	MV06L4	MV08L2	MV08L3
Superior / Anterior / Left	11.3	1.9	27.2	6.0	18.8	10.3
Superior / Anterior / Semi	12.9	0.4	15.8	13.7	21.8	8.6
Superior / Anterior / Right	21.2	1.2	3.1	8.4	5.3	12.4
Superior / Centre / Left	10.7	1.6	12.2	16.8	15.0	20.9
Superior / Centre / Semi	6.9	1.6	5.3	6.5	38.4	7.5
Superior / Centre / Right	16.9	4.3	3.4	3.2	17.1	6.6
Superior / Posterior / Left	8.7	3.2	11.6	7.7	18.1	12.1
Superior / Posterior / Semi	5.7	1.1	14.0	7.6	16.7	20.6
Superior / Posterior / Right	9.3	11.7	4.2	9.8	3.8	10.7
Middle / Anterior / Left	7.0	2.8	7.6	0.7	6.3	8.2
Middle / Anterior / Semi	1.2	0.7	5.0	4.2	17.6	4.2
Middle / Anterior / Right	12.1	0.9	7.4	7.3	11.7	3.9
Middle / Centre / Left	3.9	1.1	1.7	2.1	10.3	7.2
Middle / Centre / Semi	4.2	2.7	1.2	0.3	0.6	3.5
Middle / Centre / Right	7.2	2.0	1.6	3.0	4.6	3.4
Middle / Posterior / Left	10.7	0.8	2.9	12.9	15.0	6.8
Middle / Posterior / Semi	6.3	2.8	4.8	3.8	9.2	9.7
Middle / Posterior / Right	6.9	1.2	4.7	7.7	4.4	11.9
Inferior / Anterior / Left	6.6	4.9	8.1	7.3	19.4	9.3
Inferior / Anterior / Semi	16.6	14.0	16.1	38.0	12.7	6.0
Inferior / Anterior / Right	17.0	27.9	19.5	18.9	15.7	6.8
Inferior / Centre / Left	13.6	6.8	13.0	4.3	17.4	14.6
Inferior / Centre / Semi	2.9	15.0	11.7	21.5	33.0	5.0
Inferior / Centre / Right	10.5	28.4	8.6	9.9	13.2	22.4
Inferior / Posterior / Left	14.8	17.9	13.5	3.2	19.7	11.9
Inferior / Posterior / Semi	18.8	8.8	14.0	18.3	16.0	15.2
Inferior / Posterior / Right	9.4	11.6	5.4	3.5	43.1	11.2
Mean ± Standard Deviation	10.1 ± 5.0	6.6 ± 7.8	9.0 ± 6.2	9.1 ± 8.0	15.7 ± 9.8	10.0 ± 5.1

Colour scale: 0 – 5 – 10 – 15 – 20 – 25 – 30 – 35 – 40 – 45

Supplementary Figure G: Coefficient of variation of local maximum principal strain (ε_{p1}). Each column represents a different vertebra, and each row represents a probes XYZ location (shown in legend). The last row shows the average coefficient of variation across the probes and the standard deviation. The scale of the heatmap is from 0% to 45%. Numbers shown in red represent probes that have elements located within a lesion.

*Supplementary Table B: Mechanical results for both intra- (top) and inter- (bottom) operator assessments. Each row is the average and standard deviation for the three comparisons of the vertebra, with the average and standard deviation across all vertebrae calculated. *, **, *** represent significant differences between the metrics for the intra- and the inter-operator assessments with $p<0.05$, $p<0.01$ or $p<0.001$, respectively. The last row for both intra- and inter- shows the precision error (%) for each metric.*

Supplementary Table B

Intra-Operator	Vertebrae Type	Mean Ultimate Force (kN)**	Mean Ultimate Stress (MPa)**	Mean Stiffness (kN/mm) **	Mean Apparent Modulus (MPa) **
MV04_L4	Control	1.7 ± 0.0	1.4 ± 0.0	18.2 ± 0.5	426.6 ± 9.3
MV04_L5	Lytic	4.1 ± 0.1	2.8 ± 0.0	47.8 ± 1.0	879.7 ± 19.2
MV06_L3	Lytic	2.9 ± 0.0	2.7 ± 0.0	34.8 ± 0.4	875.1 ± 10.2
MV06_L4	Control	2.5 ± 0.0	2.5 ± 0.0	28.9 ± 0.6	809.2 ± 15.4
MV08_L2	Lytic	2.7 ± 0.0	1.9 ± 0.0	31.3 ± 1.0	557.4 ± 17.5
MV08_L3	Control	2.2 ± 0.0	1.9 ± 0.0	25.7 ± 0.3	574.8 ± 7.3
**Mean ± Standard Deviation**		**2.7 ± 0.7**	**2.2 ± 0.5**	**31.8 ± 9.1**	**687.2 ± 175.4**
**Precision Error (%)**		**1.5**	**1.3**	**2.1**	**2.1**

Inter-Operator	Vertebrae Type	Mean Ultimate Force (kN)**	Mean Ultimate Stress (MPa)**	Mean Stiffness (kN/mm) **	Mean Apparent Modulus (MPa) **
MV04_L4	Control	1.7 ± 0.0	1.4 ± 0.0	17.8 ± 0.2	417.0 ± 1.8
MV04_L5	Lytic	4.0 ± 0.1	2.7 ± 0.0	47.0 ± 0.5	860.6 ± 12.4
MV06_L3	Lytic	2.7 ± 0.1	2.7 ± 0.1	33.6 ± 1.3	850.9 ± 25.8
MV06_L4	Control	2.5 ± 0.1	2.5 ± 0.1	27.9 ± 1.2	789.8 ± 19.1
MV08_L2	Lytic	2.6 ± 0.1	1.9 ± 0.0	30.8 ± 0.6	552.4 ± 0.9
MV08_L3	Control	2.1 ± 0.1	1.8 ± 0.1	24.5 ± 1.2	556.3 ± 19.5
**Mean ± Standard Deviation**		**2.6 ± 0.7**	**2.2 ± 0.5**	**30.3 ± 9.0**	**671.1 ± 170.4**
**Precision Error (%)**		**3.6**	**2.5**	**3.3**	**2.3**

Supplementary Table C: Relative difference of mechanical metrics for both intra- (top) and inter- (bottom) operator assessments. Each row is the relative difference for the three comparisons of the vertebra, with the average and standard deviation across all vertebrae in the final row.

Supplementary Table C

Intra-Operator	Ultimate Force Relative Difference (%)	Ultimate Stress Relative Difference (%)	Stiffness Relative Difference (%)	Apparent Modulus Relative Difference (%)
MV04_L4	2.8	2.6	4.0	3.5
MV04_L5	3.4	2.6	3.4	3.6
MV06_L3	2.0	1.9	1.8	1.9
MV06_L4	2.2	2.2	3.1	3.0
MV08_L2	1.5	1.1	5.2	5.0
MV08_L3	1.9	1.7	2.0	2.0
**Mean ± Standard Deviation**	**2.3 ± 0.6**	**2.0 ± 0.6**	**3.3 ± 1.2**	**3.2 ± 1.1**
Inter-Operator	Ultimate Force Relative Difference (%)	Ultimate Stress Relative Difference (%)	Stiffness Relative Difference (%)	Apparent Modulus Relative Difference (%)
MV04_L4	3.0	2.1	2.2	1.8
MV04_L5	2.9	1.8	1.4	2.3
MV06_L3	7.4	4.2	6.4	4.5
MV06_L4	4.6	3.9	6.9	3.9
MV08_L2	3.1	1.9	2.9	0.2
MV08_L3	9.6	6.9	7.9	5.6
**Mean ± Standard Deviation**	**5.1 ± 2.6**	**3.5 ± 1.8**	**4.6 ± 2.5**	**3.1 ± 1.8**
